# Tracheomediastinal fistula induced by concurrent chemoradiotherapy in small cell lung cancer: A case report and literature review

**DOI:** 10.1111/1759-7714.15270

**Published:** 2024-03-25

**Authors:** Yoshihiro Yamamoto, Daisuke Shibahara, Taro Mori, Kohei Otsubo, Yoshimasa Shiraishi, Yasuto Yoneshima, Eiji Iwama, Kentaro Tanaka, Yoshinao Oda, Isamu Okamoto

**Affiliations:** ^1^ Department of Respiratory Medicine, Graduate School of Medical Sciences Kyushu University Fukuoka Japan; ^2^ Department of Anatomic Pathology, Graduate School of Medical Sciences Kyushu University Fukuoka Japan

**Keywords:** bronchomediastinal fistula, chemoradiotherapy, lymph node, mediastinitis, tracheomediastinal fistula

## Abstract

Tracheomediastinal fistula is a rare but life‐threatening complication of cancer. We report a case of tracheomediastinal fistula induced by concurrent chemoradiotherapy in limited stage small cell lung cancer. Despite the treatment response, the metastatic paratracheal lymph node increased gradually during concurrent chemoradiotherapy, resulting in the occurrence of tracheomediastinal fistula and mediastinitis. Without any surgical intervention, the patient achieved successful recovery from mediastinitis through antibiotic treatment, although the tracheomediastinal fistula remained open. In this report, we also review previous studies of tracheomediastinal and bronchomediastinal fistulas and summarize the clinical features.

## Graphical abstract

Tracheomediatinal fistula is a rare but life‐threatening complication of cancer. We report a case of tracheomediastinal fistula induced by concurrent chemoradiotherapy in limited stage small cell lung cancer. The metastatic paratracheal lymph node enlarged and subsequently perforated.

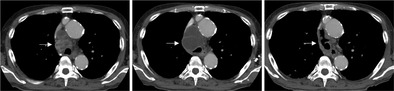

## INTRODUCTION

Small cell lung cancer is a highly aggressive tumor with a poor prognosis, although it initially responds to chemotherapy or chemoradiotherapy.^1^ Concurrent chemoradiotherapy stands as the cornerstone for managing unresectable localized limited‐stage small‐cell lung cancer (LD‐SCLC). Here, we experienced a case of tracheomediastinal fistula induced by concurrent chemoradiotherapy in LD‐SCLC. Tracheomediastinal fistula is a life‐threatening and very rare complication of cancer. Consequently, previous reports have been limited primarily to case studies and the characteristics remain largely unknown.

## CASE REPORT

**FIGURE 1:**
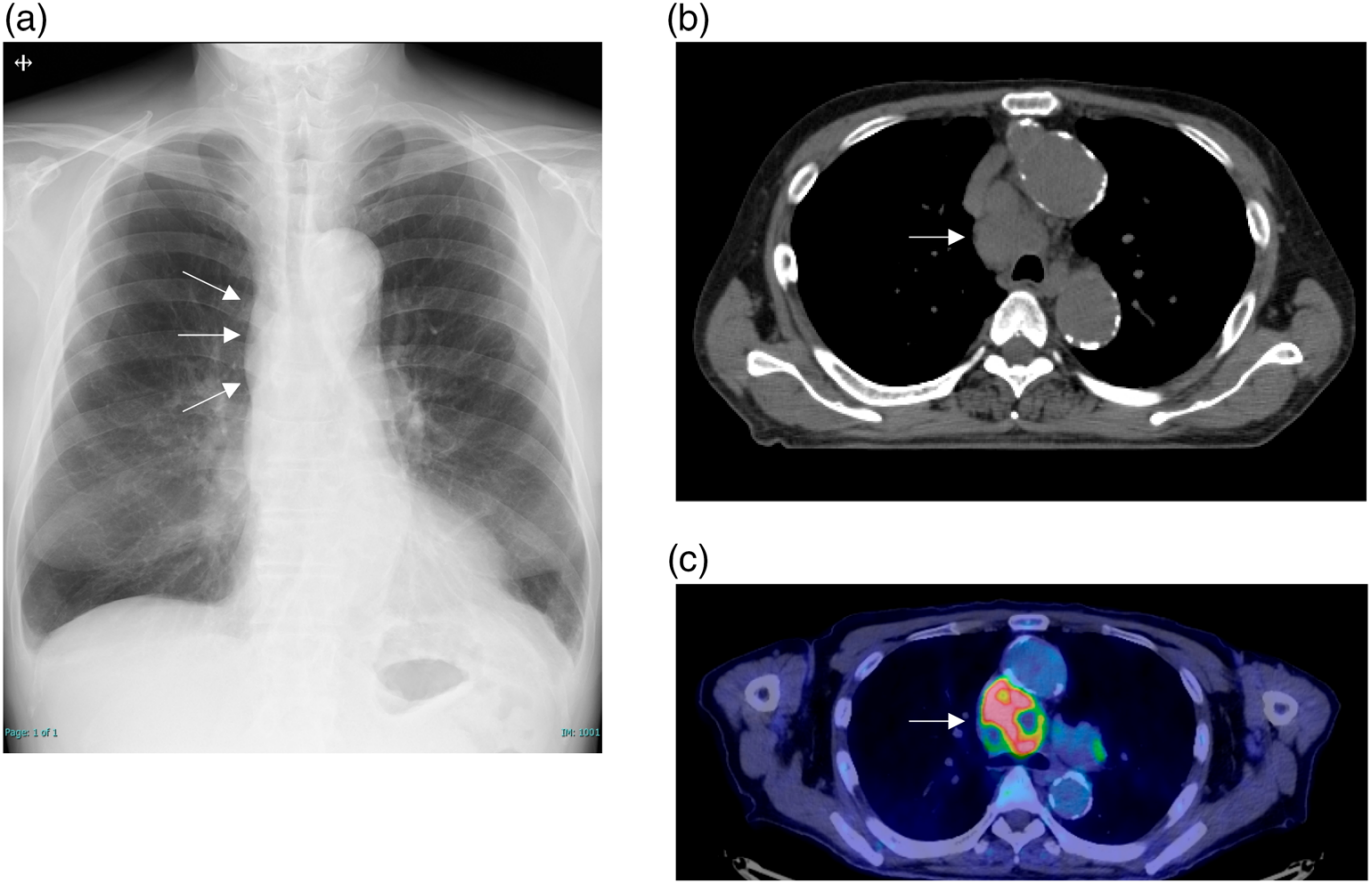
Radiological imaging at initial diagnosis. (a) A chest x‐ray showed an enlarged hilar shadow on the right side. (b) A computed tomography showed an enlarged right anterior paratracheal lymph node. (c) 18F‐fluorodeoxyglucose‐positron emission tomography showed a highly enhanced signal in an enlarged paratracheal lymph node. White arrow: metastatic paratracheal lymph node.

**FIGURE 2:**
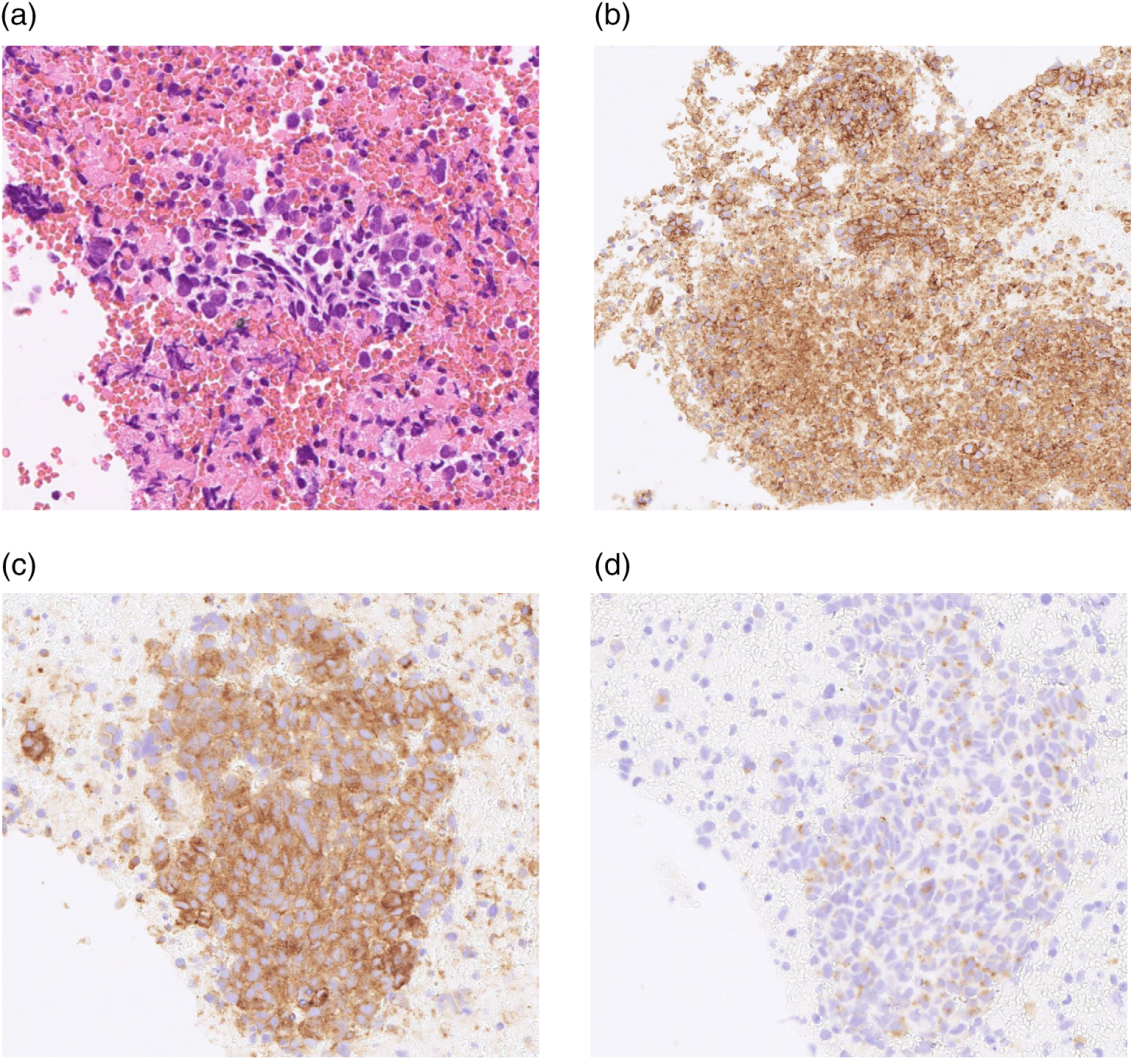
Pathological and immunohistochemical examination. (a) Tumor cells displayed a proliferation of atypical cells with hyperchromatic nuclei and scant cytoplasm, accompanied by massive necrotic tissue (hematoxylin and eosin). (b–d) Tumor cells showed positive staining for CD56 (b), synaptophysin (c), and chromogranin A (d).

A 72‐year‐old male patient presented at our hospital with complaints of dysphagia and blurred vision. A chest x‐ray and computed tomography (CT) revealed no evident tumorous lesions in the lung fields, although a 40 mm enlarged paratracheal lymph node was detected (Figure 1a,b). 18F‐fluorodeoxyglucose‐positron emission tomography (18F‐FDG‐PET) showed abnormal accumulation in the corresponding lymph node, with no evidence in other lymph nodes or distant metastases (Figure 1c). Endobronchial ultrasound‐guided transbronchial needle aspiration (EBUS‐TBNA) was performed, and histopathological examination revealed necrotic lesions with infiltration of atypical cells positive for CD56, synaptophysin, and chromogranin A (Figure 2), leading to a diagnosis of small cell lung carcinoma (cT0N2M0 stage IIIA). As the acetylcholine receptor antibody titer was positive at 31.5 nmol/L, the patient was concomitantly diagnosed with moderate generalized myasthenia gravis (defined as Myasthenia Gravis Foundation of America classification of IIIb). In order to treat dysphagia, prednisolone and pyridostigmine were administered, leading to an amelioration of his symptoms.

**FIGURE 3:**
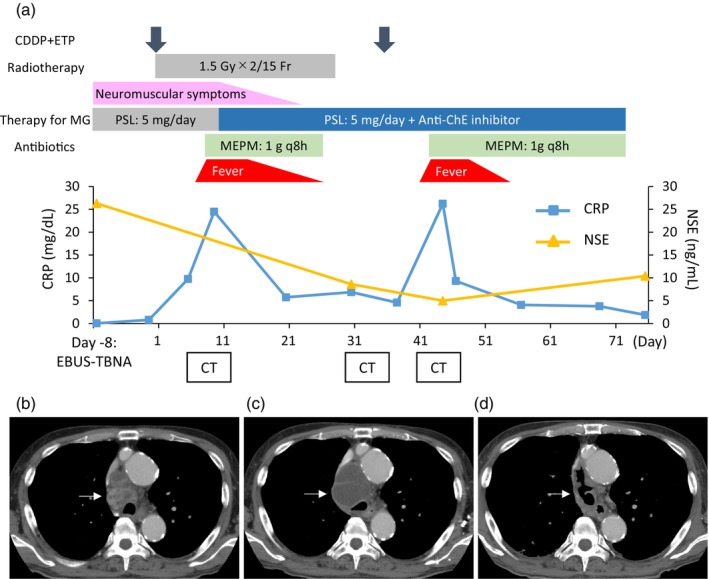
Time course of treatment, serum markers, and computed tomography. (a) Time course of treatment and the change of serum markers. (b) An enlarged paratracheal lymph node (55 mm) at day 8 after initiation of chemoradiotherapy when febrile neutropenia occurred. (c) A further enlarged paratracheal lymph node (73 mm) at day 33 after initiation of chemoradiotherapy. (d) Fistula between the paratracheal lymph node and trachea at day 44. White arrow: metastatic paratracheal lymph node. CDDP, cisplatin; CRP, C reactive protein; CT, computed tomography; EBUS‐TBNA, endobronchial ultrasound‐guided transbronchial needle aspiration; ETP, etoposide; MEPM: meropenem; MG, myasthenia gravis; NSE, neuron‐specific enolase; PSL, prednisolone.

Concurrent chemoradiotherapy with cisplatin and etoposide along with accelerated hyperfractionated radiotherapy (45 Gy) was administered. On day 7 of the first course, the patient exhibited a fever of 37.9°C, neutropenia (519/μL), and elevated inflammatory markers (C reactive protein [CRP] 16.91 mg/dL), leading to a diagnosis of febrile neutropenia (Figure 3a). A chest CT revealed an enlargement of the paratracheal lymph node to 55 mm (Figure 3b). A 14‐day course of antibiotic treatment ameliorated his symptoms and reduced inflammatory markers, despite a continued increase in the lymph node size to 73 mm (Figure 3c). The enlarged lymph node exhibited homogenous low attenuation with no definite contrast enhancement in the adjacent mediastinal fat or fluid collections. Serum neuron‐specific enolase (NSE) decreased (Figure 3a). We decided to continue the chemotherapy treatment at a reduced dosage, and introduced pegfilgrastim to prevent febrile neutropenia. On day 9 of the second course, the patient exhibited a fever of 38.9°C and increased sputum production. Blood tests showed neutropenia (60/μL) and elevated inflammatory markers (CRP 26.23 mg/dL), leading to a diagnosis of recurrent febrile neutropenia (Figure 3a). A chest CT revealed a reduction in the size of the paratracheal lymph node to 54 mm with intraluminal cavitation, which perforated into the trachea (Figure 3d). In addition, heterogenous contrast enhancement was observed in the mediastinal fat on chest CT. Taken together, we diagnosed the patient with mediastinitis subsequent to the formation of a tracheomediastinal fistula. Although additional intervention such as surgery or stent placement was considered, the control of infection was prioritized. Despite conducting multiple assessments of blood and sputum cultures, blood cultures were all negative and sputum culture revealed only oral microbiota. Due to the development of febrile neutropenia, the possibility of *Pseudomonas Aeruginosa* infection could not be excluded. Consequently, in managing febrile neutropenia and mediastinitis, the patient received antibiotic treatment with meropenem for 31 days, resulting in successful recovery despite the persistent fistula. Given the high risk of severe infection, continuation of chemotherapy was not performed. Four months after the initiation of treatment, there was no evident recurrence of cancer; thus, stent placement was considered to repair the fistula.

## DISCUSSION

Concurrent chemoradiotherapy dramatically reduces tumor volume of SCLC over a short period of time, and in one study 77% of LD‐SCLC patients with stage III disease showed radiological tumor response (defined as complete or partial response).^2^ In the present case, the metastatic paratracheal lymph node exhibited progressive enlargement during concurrent chemoradiotherapy, which made it difficult to distinguish treatment response from tumor progression or abscess formation. Serum NSE decreased throughout the treatment period, indicating a favorable response to chemoradiotherapy, as NSE correlates with response to treatment.^3^ Furthermore, the initially heterogeneous enhancement of the enlarged lymph node transformed into homogeneous low attenuation without adjacent fat enhancement. This change was accompanied by concomitant improvements in inflammatory markers, indicating a reduced likelihood of abscess or mediastinitis.^4^ We determined that the effects of chemoradiotherapy induced necrosis in tumor cells, contributing to the enlargement of the paratracheal lymph node. Pathological examination could differentiate between them, although a re‐evaluation through EBUS‐TBNA was not performed as the procedure was considered to be high‐risk for perforation and infection. PET, magnetic resonance imaging or serum IL‐8 might contribute to identify these differential diagnosis.^5^, ^6^ A more definitive diagnostic tool is warranted, while a comprehensive diagnosis based on physical examination, imaging findings, as well as serum inflammatory and tumor markers has to be made.

Adverse events associated with concurrent chemoradiotherapy include myelosuppression, esophagitis, and pneumonitis, while tracheomediastinal fistula is a very rare complication. To the best of our knowledge, there have been no reported cases of tracheomediastinal fistula formation during chemoradiotherapy for small cell lung cancer. In order to identify common features, risk factors, and treatment strategies for tracheomediastinal fistula, we searched and reviewed relevant case reports from 2013 to 2023 using keywords such as “mediastinal,” “fistula,” and “cancer” on PubMed. There were eight case reports of tracheomediastinal and bronchomediastinal fistulas available in English, which are summarized in Table 1 including the present case.^7^, ^8^, ^9^, ^10^, ^11^, ^12^, ^13^, ^14^ The median age was 60 (range: 22–75), and seven out of nine were men. The underlying diseases included six cases of lung cancer, and two cases of lymphoma. Among nine cases, three cases were treated with chemoradiotherapy and two cases were treated with vascular endothelial growth factor inhibitor (bevacizumab). Necrosis induced by radiotherapy or delayed wound healing by bevacizumab treatment have been reported as the risk factors for fistula formation in gastrointestinal cancers.^15^, ^16^, ^17^ The duration from the initial treatment of tumors to fistula formation varied from 2 weeks to 3 months. Intriguingly, seven out of nine cases developed fistula on the anterior or lateral wall of the trachea with better prognosis, while fistulas located on the posterior walls of mainstem bronchus were associated with a poor prognosis. This might be because the posterior walls of the lower trachea and mainstem bronchus are contiguous with the sparse connective tissue known as the “danger space,” easily facilitating the spread of infection.^18^ Most reports lacked detailed descriptions of necrotic features and tumor size, while the present case exhibited necrosis in a huge mediastinal lymph node. Necrotic features and tumor size could be the risk factors of fistula formation. In the case reports reviewed, the use of steroids was not documented; however, numerous reports highlight the potential risks associated with steroid use in perforation of intestine, diverticula, and esophagus;^19^, ^20^, ^21^ thus, prednisolone for treatment of myasthenia gravis might contribute to delayed healing in damaged areas, resulting in tracheomediastinal fistula.

**TABLE 1:** Summary of previously reported cases of tracheomediastinal and bronchomediastinal fistulas.

Case (#Ref)	Age	Sex	Underlying disease	Location of paratracheal lymph node	Site of perforation	Treatment before fistula formation	Time to fistula formation	Management of fistula	Outcome
1 (#7)	60	M	Non‐Hodgkin's lymphoma	Right	Right anterior tracheal wall	Non	0 day	Uncovered metallic stent	Discharged
2 (#8)	47	M	Lung adenocarcinoma	Right posterior‐lateral	Right posterior‐lateral tracheal wall	Chemotherapy (CDDP + GEM)	Three months after chemotherapy	Argon plasma coagulation	No data
3 (#9)	58	M	Lung adenocarcinoma	Right posterior‐lateral	Right posterior‐lateral tracheal wall	Bevacizumab after chemoradiotherapy (CBDCA + PTX + ? Gy)	Six weeks after bevacizumab	Covered stent	Discharged
4 (#10)	60	M	Lung squamous carcinoma	Right	Posterior wall of right mainstem bronchus	Chemotherapy (CBDCA + GEM)	After one cycle	Non	Died
5 (#11)	63	F	Lung adenocarcinoma	Right	Right anterior‐lateral tracheal wall	Chemotherapy (pemetrexed + bevacizumab)	Two weeks after chemotherapy	Antibiotics	Palliative care
6 (#12)	75	M	Lung adenocarcinoma	Left	Posterior wall of left mainstem bronchus	Durvalumab after chemoradiotherapy (CBDCA + PTX + 60 Gy/30 Fr)	After five cycles	Non	Palliative care
7 (#13)	69	F	Solitary pulmonary nodule	Right	Right anterior‐lateral tracheal wall	Non	Six weeks after EBUS‐TBNA	Antibiotics, surgical debridement	Discharged
8 (#14)	22	M	B cell lymphoma	Anterior	Left anterior‐lateral tracheal wall	Chemotherapy (REPOCH)	After four cycles	No data	No data
9 (our case)	72	M	Small cell lung carcinoma	Anterior	Right anterior tracheal wall	Chemoradiotherapy (CDDP + ETP + 45 Gy/30 Fr)	After two cycles	Antibiotics	Discharged

Abbreviations: CBDCA, carboplatin; CDDP, cisplatin; EBUS‐TBNA, endobronchial ultrasound‐guided transbronchial needle aspiration; EPOCH, rituximab‐etoposide‐prednisolone‐vincristine‐cyclophosphamide‐hydroxydanorubicin; ETP, etoposide; Fr, fraction; GEM, gemcitabine; PTX, paclitaxel.

The treatment strategy for tracheomediastinal fistula is very limited. Surgery, stent placement,^7^, ^9^ and argon plasma coagulation^8^ can be performed to treat the fistula, although most patients might opt for palliative care because of their general condition (Table 1). Patients who underwent interventions such as stent placement or surgical debridement exhibited better outcomes in the reviewed case reports. As tracheomediastinal fistula and subsequent mediastinitis are critical complications, early detection and immediate antibiotic treatment are essential for a favorable outcome. To administer appropriate antibiotic treatment as soon as possible when mediastinitis occurs, careful and close observation is necessary during chemoradiotherapy in small cell lung cancer with metastatic paratracheal lymph nodes. Additionally, patients with small cell lung cancer have a poor prognosis and chemoradiotherapy is the only curative treatment option available for inoperable LD‐SCLC. Therefore, despite the risk of tracheomediastinal fistula development in an enlarged lymph node, chemoradiotherapy may be advisable to achieve a cure for LD‐SCLC.

In summary, we describe a case of tracheomediastinal fistula induced by concurrent chemoradiotherapy in small cell lung cancer. As tracheomediastinal fistula is a life‐threatening complication, we should be aware of the risks and perform careful and close observation during concurrent chemoradiotherapy.

## AUTHOR CONTRIBUTIONS

All authors had full access to the data in the study and take responsibility for the integrity of the data and the accuracy of the data analysis. Conceptualization: Yoshihiro Yamamoto, Daisuke Shibahara and Isamu Okamoto. Methodology: Yoshihiro Yamamoto and Daisuke Shibahara. Investigation: Yoshihiro Yamamoto and Daisuke Shibahara. Resources: Yoshihiro Yamamoto, Daisuke Shibahara and Taro Mori. Writing‐original draft: Yoshihiro Yamamoto and Daisuke Shibahara. Writing‐review and editing: Yoshihiro Yamamoto, Daisuke Shibahara and Isamu Okamoto. Visualization: Yoshihiro Yamamoto, Daisuke Shibahara and Taro Mori. Supervision: Daisuke Shibahara, Kohei Otsubo, Yoshimasa Shiraishi, Yoshihiro Yamamoto, Eiji Iwama, Kentaro Tanaka, Yoshinao Oda, and Isamu Okamoto.

## CONFLICT OF INTEREST STATEMENT

Isamu Okamoto reports a relationship with Chugai Pharmaceutical Co Ltd that includes funding grants and speaking and lecture fees. Isamu Okamoto reports a relationship with AstraZeneca Pharmaceuticals LP that includes: funding grants and speaking and lecture fees. Isamu Okamoto reports a relationship with Daiichi Sankyo Co Ltd that includes: funding grants. Isamu Okamoto reports a relationship with Merck & Co Inc that includes: funding grants. Isamu Okamoto reports a relationship with Takeda Pharmaceutical Company Limited that includes: funding grants and speaking and lecture fees. Isamu Okamoto reports a relationship with Eli Lilly Japan KK that includes: funding grants and speaking and lecture fees. Isamu Okamoto reports a relationship with Ono Pharmaceutical Co Ltd that includes: funding grants and speaking and lecture fees. Isamu Okamoto reports a relationship with Bristol‐Myers Squibb Co that includes: funding grants and speaking and lecture fees. Isamu Okamoto reports a relationship with Taiho Pharmaceutical Co Ltd that includes: funding grants and speaking and lecture fees. Isamu Okamoto reports a relationship with Nippon Boehringer Ingelheim Co Ltd that includes: funding grants and speaking and lecture fees. Isamu Okamoto reports a relationship with Novartis Pharmaceuticals that includes: speaking and lecture fees. The authors thank both the patient and his family, and report this case with written form of consent from the patient.

## References

[1] Rudin CM , Brambilla E , Faivre‐Finn C , Sage J . Small‐cell lung cancer. Nat Rev Dis Primers. 2021;7(1):3. 10.1038/s41572-020-00235-0 33446664 PMC8177722

[2] Salem A , Mistry H , Hatton M , et al. Association of Chemoradiotherapy With Outcomes Among Patients With Stage I to II vs Stage III Small Cell Lung Cancer: Secondary Analysis of a Randomized Clinical Trial. JAMA Oncol. 2019;5(3):e185335. 10.1001/jamaoncol.2018.5335 30520977 PMC6439849

[3] Isgro MA , Bottoni P , Scatena R . Neuron‐specific enolase as a biomarker: biochemical and clinical aspects. Adv Exp Med Biol. 2015;867:125–143. 10.1007/978-94-017-7215-0_9 26530364

[4] Bou‐Assaly W , McKellop J , Mukherji S . Computed tomography imaging of acute neck inflammatory processes. World J Radiol. 2010;2(3):91–96. 10.4329/wjr.v2.i3.91 21160941 PMC2999315

[5] Kebir S , Rauschenbach L , Galldiks N , Schlaak M , Hattingen E , Landsberg J , et al. Dynamic O‐(2‐[18F]fluoroethyl)‐L‐tyrosine PET imaging for the detection of checkpoint inhibitor‐related pseudoprogression in melanoma brain metastases. Neuro Oncol. 2016;18(10):1462–1464. 10.1093/neuonc/now154 27591333 PMC5035529

[6] Sanmamed MF , Perez‐Gracia JL , Schalper KA , Fusco JP , Gonzalez A , Rodriguez‐Ruiz ME , et al. Changes in serum interleukin‐8 (IL‐8) levels reflect and predict response to anti‐PD‐1 treatment in melanoma and non‐small‐cell lung cancer patients. Ann Oncol. 2017;28(8):1988–1995. 10.1093/annonc/mdx190 28595336 PMC5834104

[7] Huang CL , Chen HC , Huang HC , Cheng CY . Tracheomediastinal fistula caused by non‐Hodgkin's lymphoma. Ann Thorac Cardiovasc Surg. 2014;20:599–601. 10.5761/atcs.cr.12.02230 23603637

[8] Ucer M , Ordu C , Pilanc KN , Dalar L . Tracheomediastinal fistula in a patient with lung adenocarcinoma and its treatment with argon plasma coagulation: a case report. Medicine. 2014;93(23):e156. 10.1097/MD.0000000000000156 25415672 PMC4616342

[9] Machuzak MS , Santacruz JF , Jaber W , Gildea TR . Malignant tracheal‐mediastinal‐parenchymal‐pleural fistula after chemoradiation plus bevacizumab: management with a Y‐silicone stent inside a metallic covered stent. J Bronchol Interv Pulmonol. 2015;22(1):85–89. 10.1097/LBR.0000000000000099 25590491

[10] Piciucchi S , Gurioli C , Barone D , Gurioli C , Bertocco M , Mengozzi M , et al. Life‐threatening bronchomediastinal fistula complicating a first cycle of chemotherapy in a stage IV NSCLC case. J Thorac Oncol. 2015;10(4):717–718. 10.1097/JTO.0000000000000373 25789836

[11] Thawani R , Thomas A , Thakur K . Tracheomediastinal Fistula: Rare Complication of Treatment with Bevacizumab. Cureus. 2018;10(4):e2419. 10.7759/cureus.2419 29872599 PMC5985921

[12] Sumi T , Ikeda T , Kure K , Yamada Y , Nakata H , Mori Y . Bronchomediastinal fistula during durvalumab therapy after chemoradiotherapy in stage III NSCLC. J Thorac Oncol. 2019;14(10):1860–1861. 10.1016/j.jtho.2019.06.003 31300340

[13] Jang JG , Ahn JH , Lee SS . Delayed onset of mediastinitis with tracheomediastinal fistula following endobronchial ultrasound‐guided transbronchial needle aspiration; a case report. Thorac Cancer. 2021;12(7):1134–1136. 10.1111/1759-7714.13888 33605045 PMC8017241

[14] Singh V , Ora M , Gambhir S . Malignant tracheoparenchymal fistula in primary mediastinal B‐cell lymphoma detected on fluorodeoxyglucose positron emission tomography/computed tomography. Indian J Nucl Med. 2022;37(4):367–369. 10.4103/ijnm.ijnm_40_22 36817202 PMC9930462

[15] Saif MW , Elfiky A , Salem RR . Gastrointestinal perforation due to bevacizumab in colorectal cancer. Ann Surg Oncol. 2007;14(6):1860–1869. 10.1245/s10434-006-9337-9 17356952

[16] Cao Y , Tan A , Gao F , Liu L , Liao C , Mo Z . A meta‐analysis of randomized controlled trials comparing chemotherapy plus bevacizumab with chemotherapy alone in metastatic colorectal cancer. Int J Colorectal Dis. 2009;24(6):677–685. 10.1007/s00384-009-0655-9 19184059

[17] Kato T , Kazama Y , Matsuura S , Nagaoka S . Surgical treatment of esophageal perforation after stereotactic body radiotherapy: a report of two cases. Int J Surg Case Rep. 2023;102:107805. 10.1016/j.ijscr.2022.107805 36502658 PMC9758521

[18] Mnatsakanian A , Minutello K , Black AC , Bordoni B . Anatomy, Head and Neck, Retropharyngeal Space. StatPearls; 2024. https://www.ncbi.nlm.nih.gov/books/NBK537044/ 30725729

[19] Moon CM , Cheon JH , Shin JK , Jeon SM , Bok HJ , Lee JH , et al. Prediction of free bowel perforation in patients with intestinal Behcet's disease using clinical and colonoscopic findings. Dig Dis Sci. 2010;55(10):2904–2911. 10.1007/s10620-009-1095-7 20094787

[20] Sainathan S , Andaz S . A systematic review of transesophageal echocardiography‐induced esophageal perforation. Echocardiography. 2013;30(8):977–983. 10.1111/echo.12290 23834425

[21] Broersen LHA , Horvath‐Puho E , Pereira AM , Erichsen R , Dekkers OM , Sorensen HT . Corticosteroid use and mortality risk in patients with perforated colonic diverticular disease: a population‐based cohort study. BMJ Open Gastroenterol. 2017;4(1):e000136. 10.1136/bmjgast-2017-000136 PMC5387955 28461904

